# Supplementary material for: CNK2 promotes cancer cell motility by mediating ARF6 activation downstream of AXL signalling
Source: Nat Commun. 2023 Jun 15;14:3560. doi: 10.1038/s41467-023-39281-z (PMC10272126; doi:10.1038/s41467-023-39281-z)

Supplementary Information

CNK2 promotes cancer cell motility by mediating ARF6 activation downstream of AXL signalling

Guillaume Serwe, David Kachaner, Jessica Gagnon, Cédric Plutoni, Driss Lajoie, Eloïse Duramé, Malha Sahmi, Damien Garrido, Martin Lefrançois, Geneviève Arseneault, Marc K. Saba-El-Leil, Sylvain Meloche, Gregory Emery, and Marc Therrien

Supplementary Figures

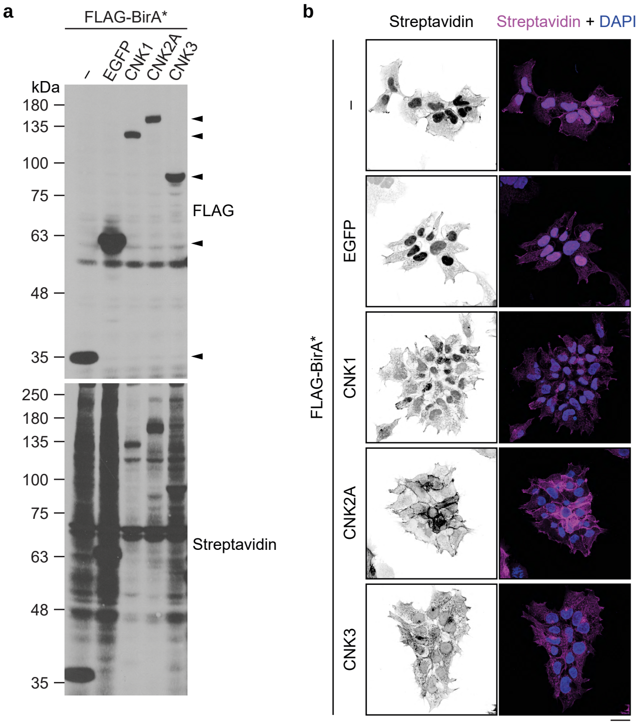

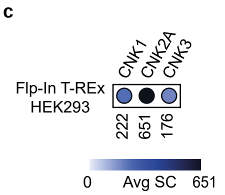

e

CNK2A

Gene ontology (GO)	Biological Process	FDR
GO:0007163	Establishment or maintenance of cell polarity	1.78e-05
GO:0007010	Cytoskeleton organization	6.70e-04
GO:0030155	Regulation of cell adhesion	3.00e-03
GO:0034330	Cell junction organization	1.41e-02

CNK3

Gene ontology (GO)	Biological Process	FDR
GO:0034330	Cell junction organization	5.86e-08
GO:0007010	Cytoskeleton organization	1.28e-07
GO:0007163	Establishment or maintenance of cell polarity	1.33e-05
GO:0051726	Regulation of cell cycle	6.06e-05
GO:0007155	Cell adhesion	5.20e-03
GO:0030155	Regulation of cell adhesion	4.18e-02

FDR from STRING network version 11.5

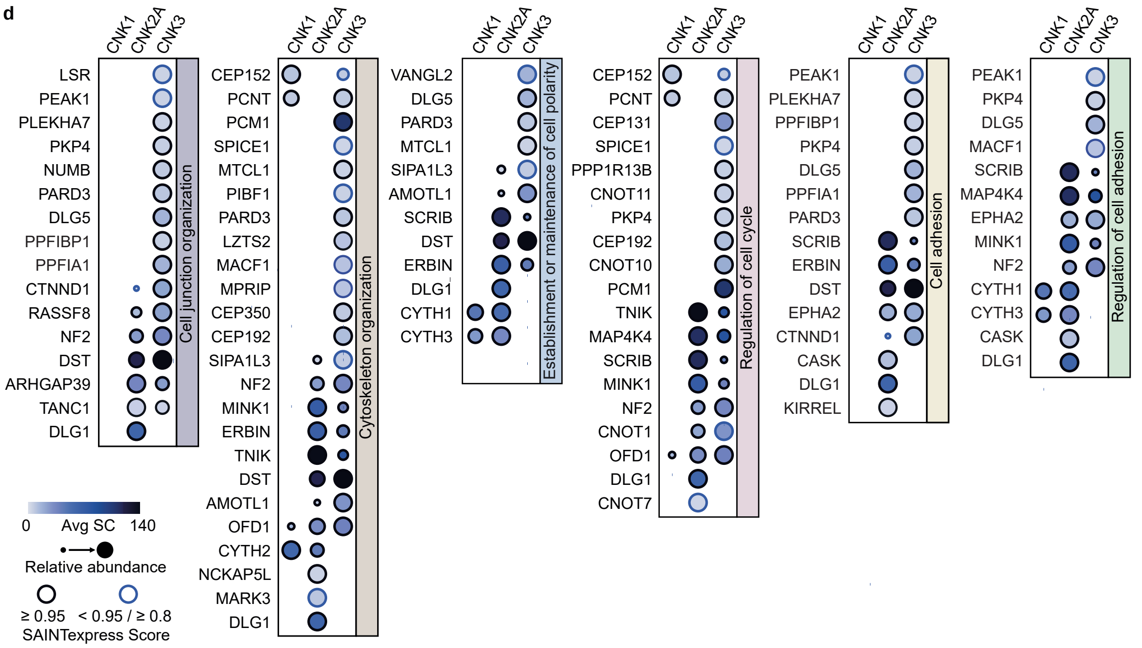

f

Percent identity matrix

	CNK1	CNK2	CNK3
**CNK1**	100.00	31.68	30.11
**CNK2**		100.00	57.55
**CNK3**			100.00

Supplementary Fig. 1: Proximal interactome of human CNKs in Flp-In T-REx HEK293 cells. **a** Immunoblot of FLAG-BirA*-tagged proteins (indicated with arrowheads) and global protein biotinylation in HEK293 Flp-In T-REx cells. **b** Representative pictures showing the localization of biotinylated proteins in HEK293 Flp-In T-REx cells. **c** Dot plot representation of the average spectral counts obtained for each of the following BioID baits: FLAG-BirA*-CNK1, -CNK2A and -CNK3 from three independent experiments. **d** Dot plot representation of the proximal interactors identified by BioID for each bait. The proximal interactors are grouped by Biological Processes (Gene Ontology) of interest. **e** Tables indicating the false discovery rate for each Biological Process. **f** Percent identity matrix, generated by Clustal2.1, revealed that CNK2 and CNK3 sequences share the highest amino acid sequence identity. Since CNK3 has the shortest protein sequence (555 amino acids), we used the first 555 amino acids of CNK1, CNK2 and CNK3 for sequence alignment. Scale bar: (**b**) 20 µm.

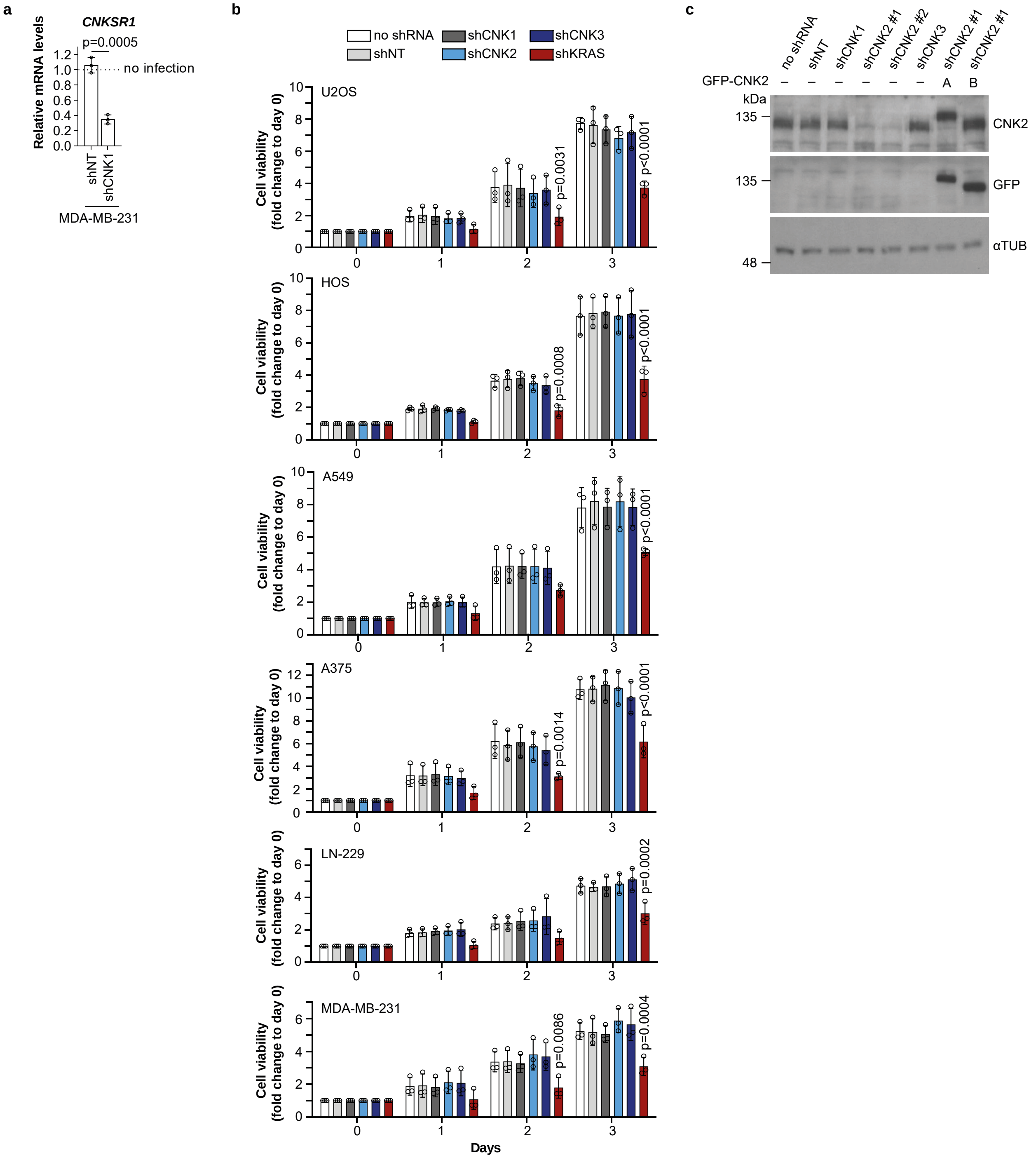

Supplementary Fig. 2: CNK2A is required for cell migration and its depletion does not impact cell viability.

a CNK1 depletion in MDA-MB-231 cells was quantified by RT-qPCR. **b** Neither CNK1, CNK2 nor CNK3 depletion affected cell proliferation or cell viability. KRAS knockdown was used as a positive control of reduced cell proliferation or viability. **c** Immunoblots from U2OS cells depleted of CNK1, CNK2 or CNK3, and expressing shRNA-insensitive GFP-CNK2A or GFP-CNK2B as indicated. Note that GFP-CNK2B has the same molecular weight as endogenous CNK2. All statistical analyses were performed on data from three independent experiments. Unpaired t-test was used in **a** and two-way ANOVA was used in **b**. Error bars correspond to mean values ± SD. Source data are provided as a Source Data file.

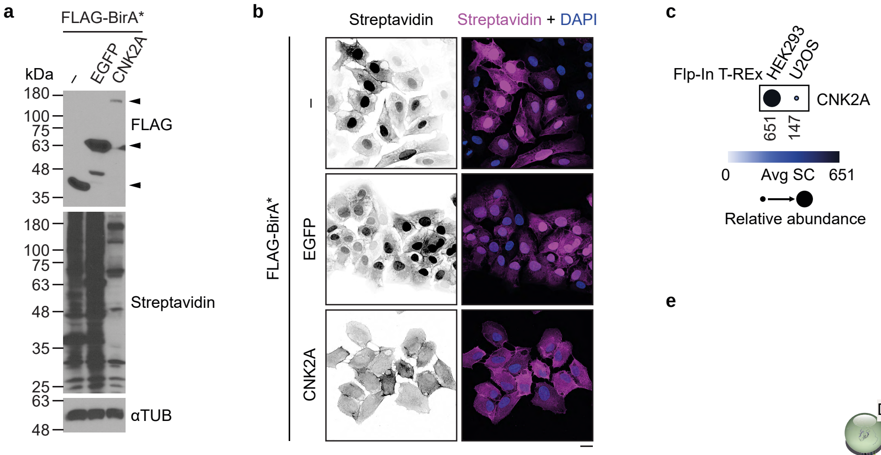

d

U2OS

Gene ontology (GO)	Biological Process	FDR
GO:0007163	Establishment or maintenance of cell polarity	1.50e-06
GO:0030155	Regulation of cell adhesion	2.37e-05
GO:0034330	Cell junction organization	1.80e-04
GO:0007155	Cell adhesion	1.20e-03
GO:0007010	Cytoskeleton organization	3.00e-03

HEK293

Gene ontology (GO)	Biological Process	FDR
GO:0007163	Establishment or maintenance of cell polarity	1.78e-05
GO:0007010	Cytoskeleton organization	6.70e-04
GO:0030155	Regulation of cell adhesion	3.00e-03
GO:0034330	Cell junction organization	1.41e-02

FDR from STRING network version 11.5

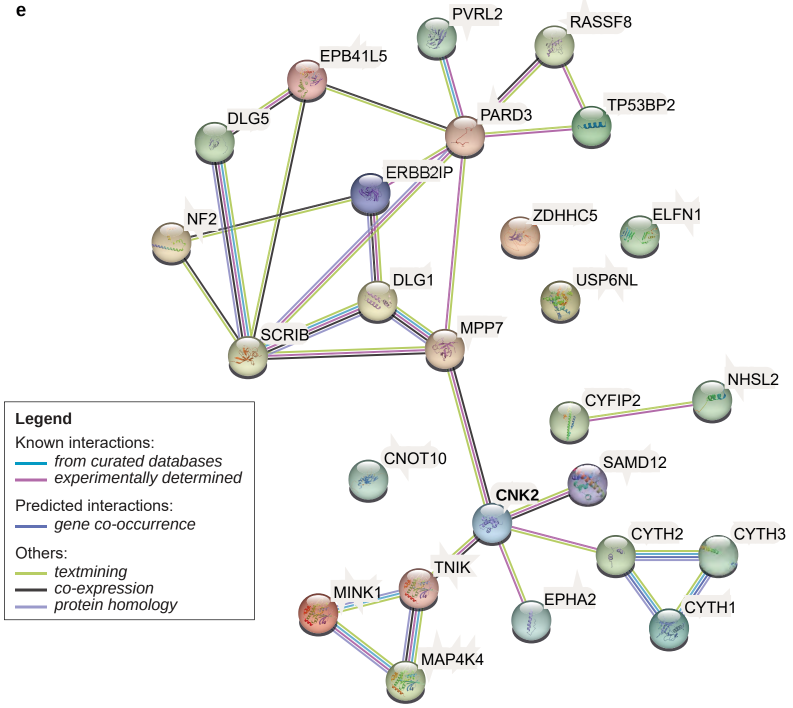

Supplementary Fig. 3: Proximal interactome of CNK2A in Flp-In T-REx U2OS cells.
a Immunoblot of FLAG-BirA*-tagged proteins (indicated with arrowheads) and global protein biotinylation in U2OS Flp-In T-REx cells. **b** Representative pictures showing the localization of biotinylated proteins in U2OS Flp-In T-REx cells. **c** Dot plot representation of the relative average spectral counts obtained by BioID for FLAG-BirA*-CNK2A bait in HEK293 and U2OS Flp-In T-REx cell lines. **d** Tables indicating the false discovery rate for each Biological Process selected to classify proximal interactors in the dot plot (**Fig. 1m**). **e** STRING network of CNK2 and its proximal interactors in U2OS cells. Scale bar: (**b**) 20 µm.

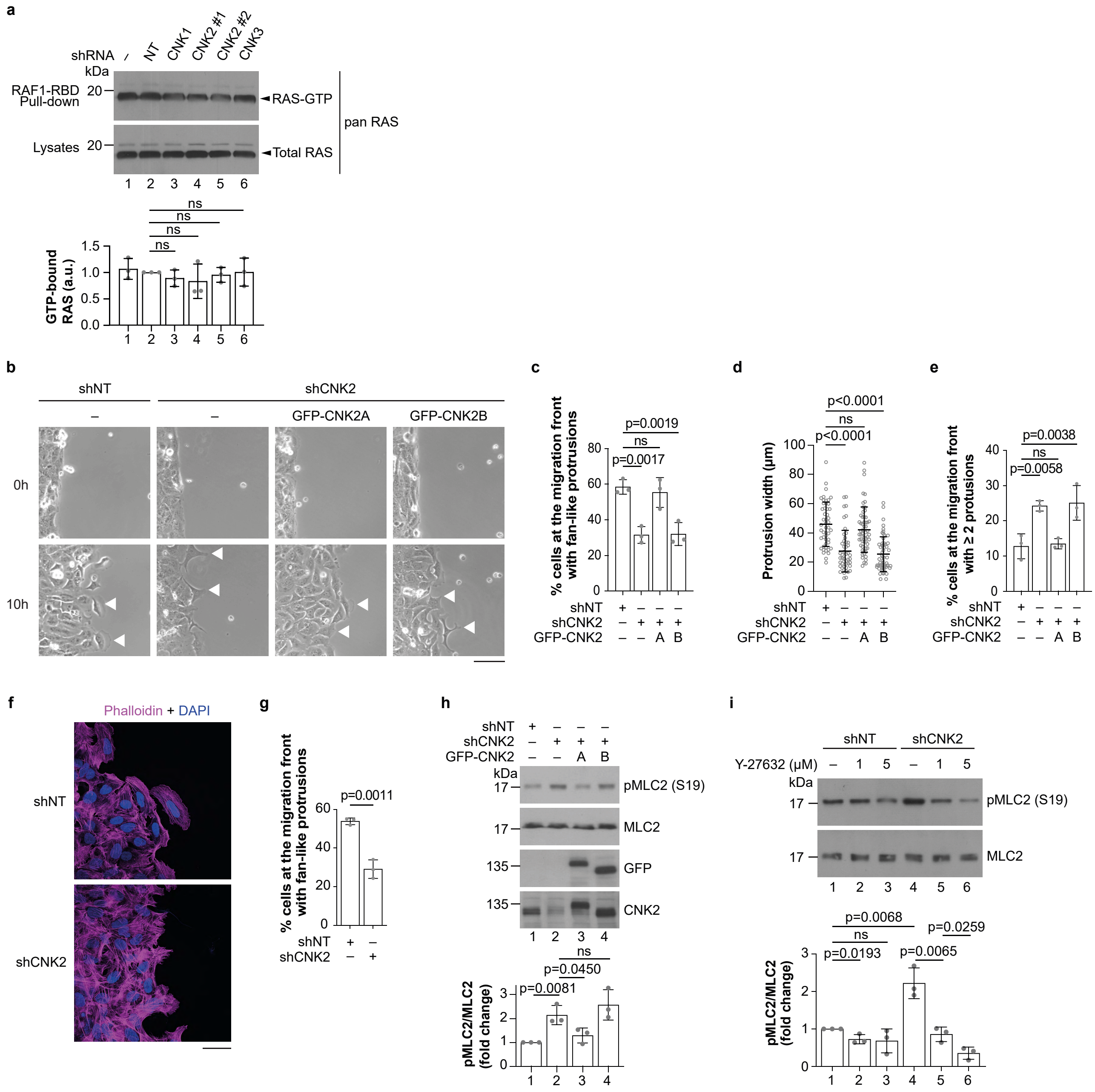

Supplementary Fig. 4: CNK2 is a positive regulator of RAC1 and a negative regulator of RHOA during migration.

a RAS-GTP was precipitated using RAF1-RBD beads. Quantification was performed as in **Fig. 2a-c**. **b** Phase contrast images of U2OS cells in wound healing-like migration assays displaying a defect in protrusion formation following CNK2 knockdown. Arrows depict protrusions of cells at the migration front. **c-e** Cells were scored for the presence of fan-like protrusions (**c**), and protrusion width was measured (**d**). The percentage of cells at the migration front with more than two protrusions was quantified (**e**). Defects in protrusion formation caused by CNK2 knockdown were rescued by GFP-CNK2A but not GFP-CNK2B. **f** Microscopy images of migrating U2OS cells depleted of CNK2 and stained with phalloidin. CNK2 depletion caused a reduction in the percentage of cells forming lamellipodia at the migration front. **g** Cells depicted in **f** were scored for the presence of fan-like protrusions. **h** CNK2 depletion in migrating U2OS cells increased levels of pMLC2. Expression of GFP-CNK2A but not GFP-CNK2B restored pMLC2 to control levels. The bar graph shows the ratio of pMLC2 to total MLC2 normalized to control cells (shNT). **i** ROCK inhibition, with 1 or 5 µM Y-27632, decreased pMLC2 levels in CNK2-depleted cells. The bar graph shows the ratio of pMLC2 to total MLC2 normalized to control cells (shNT with no treatment). All statistical analyses were performed on data from three independent experiments. One-way ANOVA was used in **a**, **c-e** and unpaired t-test was used in **g-i**. Error bars correspond to mean values ± SD; ns, not significant (P>0.05). Scale bar: (**b**) 100 µm, (**f**) 20 µm. Source data are provided as a Source Data file.

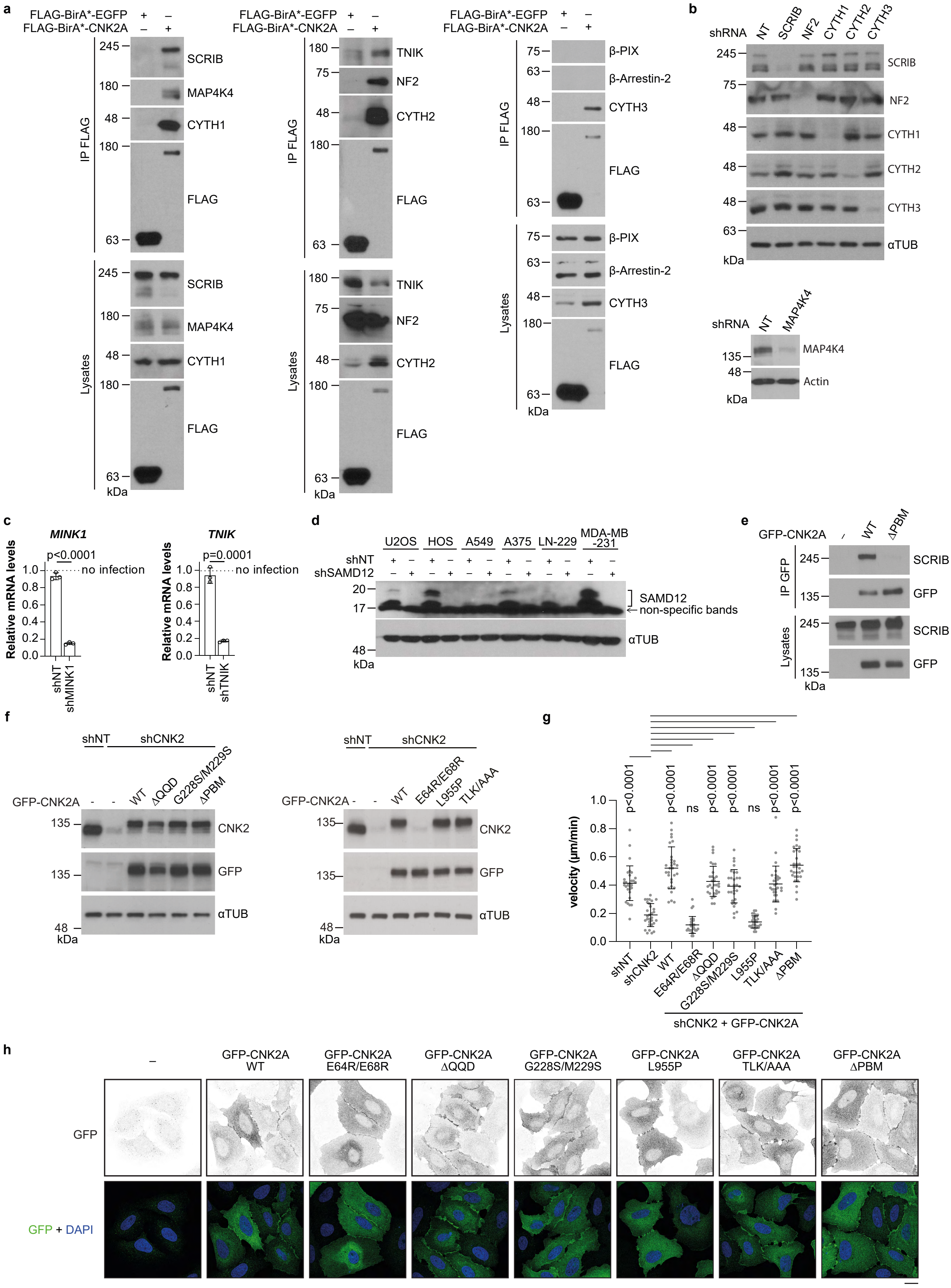

a
FLAG-BirA*-EGFP + –
FLAG-BirA*-CNK2A – +
IP FLAG
Lysates
SCRIB
MAP4K4
CYTH1
FLAG
TNIK
NF2
CYTH2
β-PIX
β-Arrestin-2
CYTH3
kDa
b
shRNA NT SCRIB NF2 CYTH1 CYTH2 CYTH3
αTUB
shRNA NT MAP4K4
Actin
c
MINK1
TNIK
Relative mRNA levels
p<0.0001
p=0.0001
no infection
shNT shMINK1
shNT shTNIK
d
U2OS HOS A549 A375 LN-229 MDA-MB -231
shNT
shSAMD12
SAMD12
non-specific bands
αTUB
e
GFP-CNK2A - WT ΔPBM
IP GFP
GFP
f
shNT shCNK2
GFP-CNK2A - - WT ΔQQD G228S/M229S ΔPBM
GFP-CNK2A - - WT E64R/E68R L955P TLK/AAA
CNK2
g
velocity (µm/min)
p<0.0001
ns
shNT shCNK2 WT E64R/E68R ΔQQD G228S/M229S L955P TLK/AAA ΔPBM
shCNK2 + GFP-CNK2A
h
–
GFP-CNK2A WT
GFP-CNK2A E64R/E68R
GFP-CNK2A ΔQQD
GFP-CNK2A G228S/M229S
GFP-CNK2A L955P
GFP-CNK2A TLK/AAA
GFP-CNK2A ΔPBM
GFP
GFP + DAPI

Supplementary Fig. 5: CYTH1, CYTH3, and SAMD12 are functional CNK2 interactors required for cell migration.

a FLAG-BirA*-CNK2A interacted with endogenous MAP4K4, TNIK, CYTH1, CYTH2, CYTH3, SCRIB and NF2 by co-IP in Flp-In T-REx U2OS cells. Note that β-PIX and β-Arrestin-2 were used as negative controls in co-IP, since they were not identified as proximal interactors in CNK2A BioIDs. Immunoblots were probed with antibodies indicated to the right. **b** Immunoblots confirming shRNA-mediated depletions of endogenous SCRIB, NF2, CYTH1,2,3 and MAP4K4 in U2OS cells. **c** TNIK or MINK1 depletion in U2OS cells was quantified by RT-qPCR. **d** shRNA-mediated depletion of endogenous SAMD12 in six cancer cell lines (specific bands are denoted by the square bracket and non-specific bands are indicated with an arrow). **e** Co-IP experiments in U2OS cells indicated that endogenous SCRIB associated with GFP-CNK2A WT, but not with GFP-CNK2A_ΔPBM. **f** Immunoblots from U2OS cells depleted of endogenous CNK2 and expressing shRNA-insensitive GFP-CNK2A WT or mutants as indicated. Note that our custom-made anti-CNK2 antibody did not detect the GFP-CNK2A E64R/E68R mutant by western blot. Equal CNK protein levels were confirmed using the anti-GFP antibody. **g** Quantification of cell velocity of the migration assays shown in **Fig. 3m**. **h** GFP staining showing that GFP-CNK2A WT and the indicated mutants all localized in the cytoplasm and at the plasma membrane in U2OS cells. All statistical analyses were performed on data from at least three independent experiments. Unpaired t-test was used in **c** and one-way ANOVA was used in **g**. Error bars correspond to mean values ± SD; ns, not significant ($P>0.05$). Scale bar: (**h**) 20 µm. Source data are provided as a Source Data file.

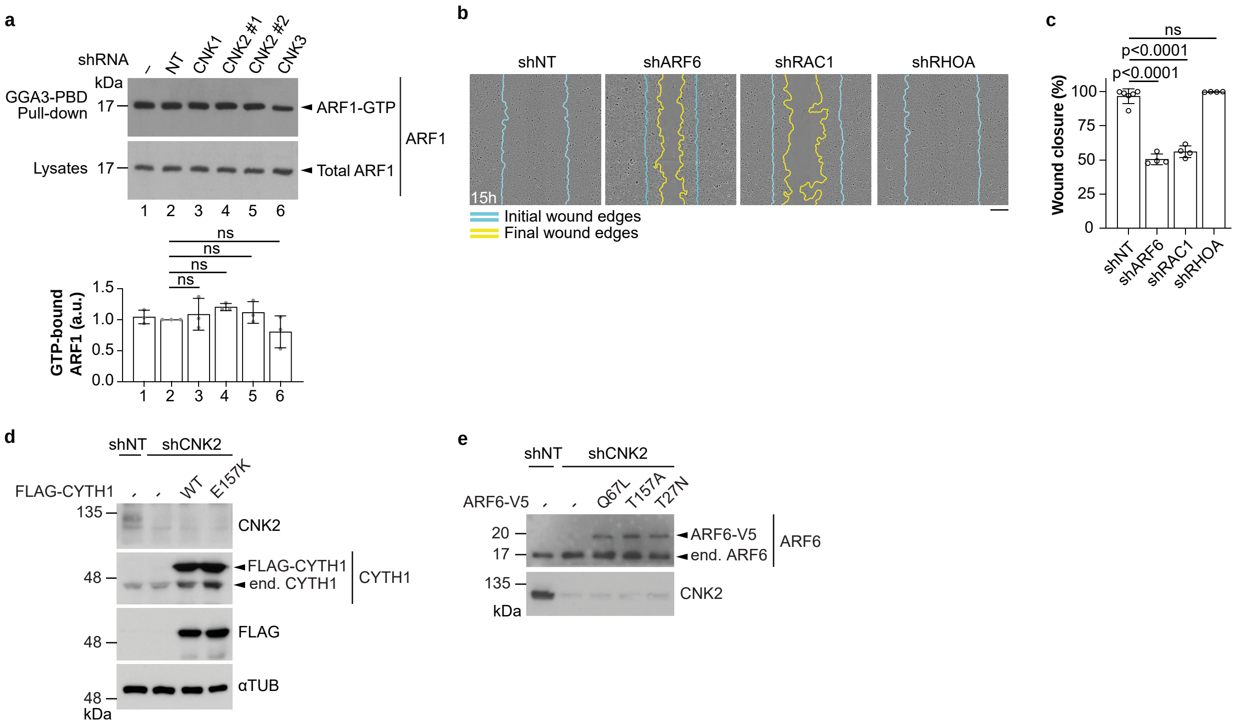

Supplementary Fig. 6: CNK2 promotes ARF6 activity upstream of RAC1 and RHOA.
a ARF1-GTP was precipitated using GGA3-PBD beads. The bar graph shows the normalized ratio of GTP-ARF1 to the amount of total ARF1. **b** Depletion of ARF6 or RAC1, but not RHOA, delayed migration of U2OS cells in wound healing assays. **c** Quantification of the migration assays shown in **b**. **d** Immunoblots from U2OS cells depleted of CNK2 and expressing FLAG-CYTH1 WT or the exchange-defective mutant (E157K). **e** Immunoblots from U2OS cells depleted of CNK2 and expressing constitutively active (Q67L), fast cycling (T157A) or dominant negative (T27N) ARF6-V5 mutants, as indicated. One-way ANOVA was performed on data from three independent experiments. Error bars correspond to mean values ± SD; ns, not significant (P>0.05). Scale bar: (**b**) 200 µm. Source data are provided as a Source Data file.

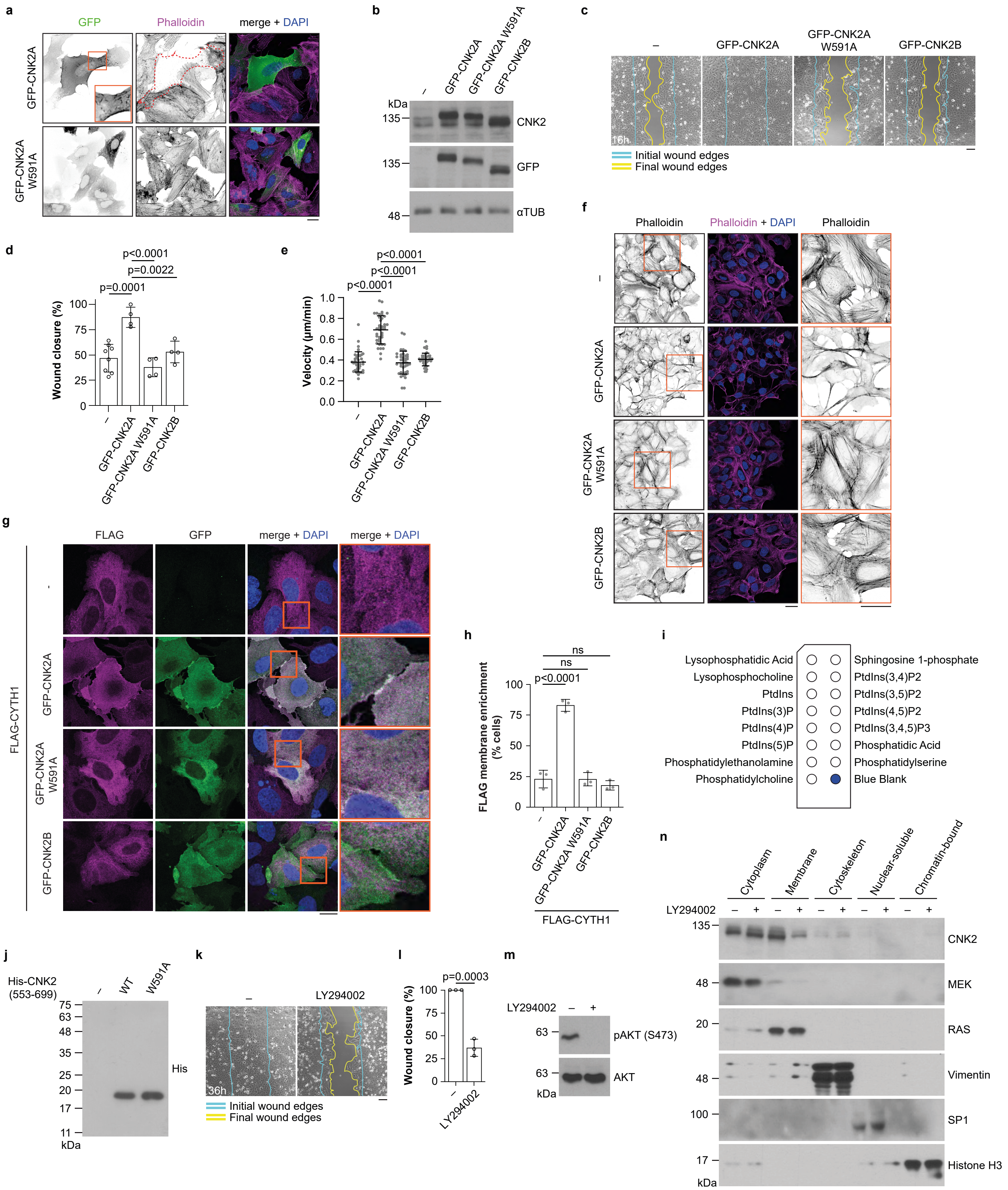

a
GFP
Phalloidin
merge + DAPI
GFP-CNK2A
GFP-CNK2A W591A
b
–
GFP-CNK2A
GFP-CNK2A W591A
GFP-CNK2B
kDa
135
CNK2
135
GFP
48
αTUB
c
–
GFP-CNK2A
GFP-CNK2A W591A
GFP-CNK2B
16h
Initial wound edges
Final wound edges
d
p<0.0001
p=0.0022
p=0.0001
Wound closure (%)
100
75
50
25
0
–
GFP-CNK2A
GFP-CNK2A W591A
GFP-CNK2B
e
p<0.0001
p<0.0001
p<0.0001
Velocity (μm/min)
1.0
0.8
0.6
0.4
0.2
0.0
–
GFP-CNK2A
GFP-CNK2A W591A
GFP-CNK2B
f
Phalloidin
Phalloidin + DAPI
Phalloidin
–
GFP-CNK2A
GFP-CNK2A W591A
GFP-CNK2B
g
FLAG
GFP
merge + DAPI
merge + DAPI
FLAG-CYTH1
-
GFP-CNK2A
GFP-CNK2A W591A
GFP-CNK2B
h
ns
ns
p<0.0001
FLAG membrane enrichment (% cells)
100
75
50
25
0
–
GFP-CNK2A
GFP-CNK2A W591A
GFP-CNK2B
FLAG-CYTH1
i
Lysophosphatidic Acid
Lysophosphocholine
PtdIns
PtdIns(3)P
PtdIns(4)P
PtdIns(5)P
Phosphatidylethanolamine
Phosphatidylcholine
Sphingosine 1-phosphate
PtdIns(3,4)P2
PtdIns(3,5)P2
PtdIns(4,5)P2
PtdIns(3,4,5)P3
Phosphatidic Acid
Phosphatidylserine
Blue Blank
j
His-CNK2 (553-699)
–
WT
W591A
75
63
48
35
25
20
17
11
kDa
His
k
–
LY294002
36h
Initial wound edges
Final wound edges
l
p=0.0003
Wound closure (%)
100
75
50
25
0
–
LY294002
m
LY294002
–
+
63
pAKT (S473)
63
AKT
kDa
n
Cytoplasm
Membrane
Cytoskeleton
Nuclear-soluble
Chromatin-bound
LY294002
– + – + – + – + – +
135
CNK2
48
MEK
20
RAS
48
Vimentin
100
SP1
17
Histone H3
kDa

Supplementary Fig. 7: CNK2 functions at the plasma membrane.

a The W591A mutation in the CNK2A PH domain prevented the localization of CNK2A at the plasma membrane. U2OS cells overexpressing GFP-CNK2A WT or GFP-CNK2A_W591A were stained with phalloidin. The dashed line outlines the GFP-positive cell. Overexpression of GFP-CNK2A resulted in the loss of filamentous actin staining. The area within the orange box is magnified in the bottom right corner of the image. **b** Immunoblots from U2OS cells overexpressing GFP-CNK2A, GFP-CNK2A_W591A or GFP-CNK2B. **c** Overexpression of GFP-CNK2A WT, but not GFP-CNK2A_W591A or GFP-CNK2B, increased migration of U2OS cells in wound healing-like assays. **d** Quantification of the migration assays shown in **c**. **e** Cell velocity was measured from the migration assay shown in **c**. 40 cells from four different experiments were tracked over a 10-hour period. **f** Overexpression of GFP-CNK2A WT, but not GFP-CNK2A_W591A or GFP-CNK2B, decreased stress fibers in U2OS migrating cells. Cells were stained with phalloidin. Areas delineated by the orange square are magnified on the right. **g** FLAG-CYTH1 staining at the plasma membrane in U2OS cells is strongly increased when co-expressed with GFP-CNK2A but not with GFP-CNK2A_W591A or GFP-CNK2B. **h** Quantification of the IF shown in **g**. At least 100 cells from three independent experiments were quantified for each condition. Each dot represents the percentage of cells displaying an enrichment of FLAG staining at the plasma membrane in one experiment. **i** Schematic representation of the phospholipids present on PIP Strips membranes. **j** Expression of the purified His-tagged versions of recombinant CNK2 protein (WT or W591A) used in the PIP Strips assays in **Fig. 5l**. **k** Treatment of U2OS with LY294002 (10 μM) delayed migration of U2OS cells in wound healing-like assays. **l** Quantification of the migration assays shown in **k**. **m** Treatment of U2OS cells with LY294002 (10 μM) strongly impaired phosphorylation of AKT on its activation site S473. **n** Fractionation of U2OS cell lysates indicated that treatment with LY294002 (10 μM) decrease the detection of endogenous CNK2 in the membrane fraction. All statistical analyses were performed on data from three independent experiments. One-way ANOVA was used in **d**, **e**, **h**, and unpaired t-test was used in **l**. Error bars correspond to mean values ± SD; ns, not significant ($P>0.05$). Scale bars: (**a**, **f**, **g**) 20 μm, (**c**, **k**) 100 μm. Source data are provided as a Source Data file.

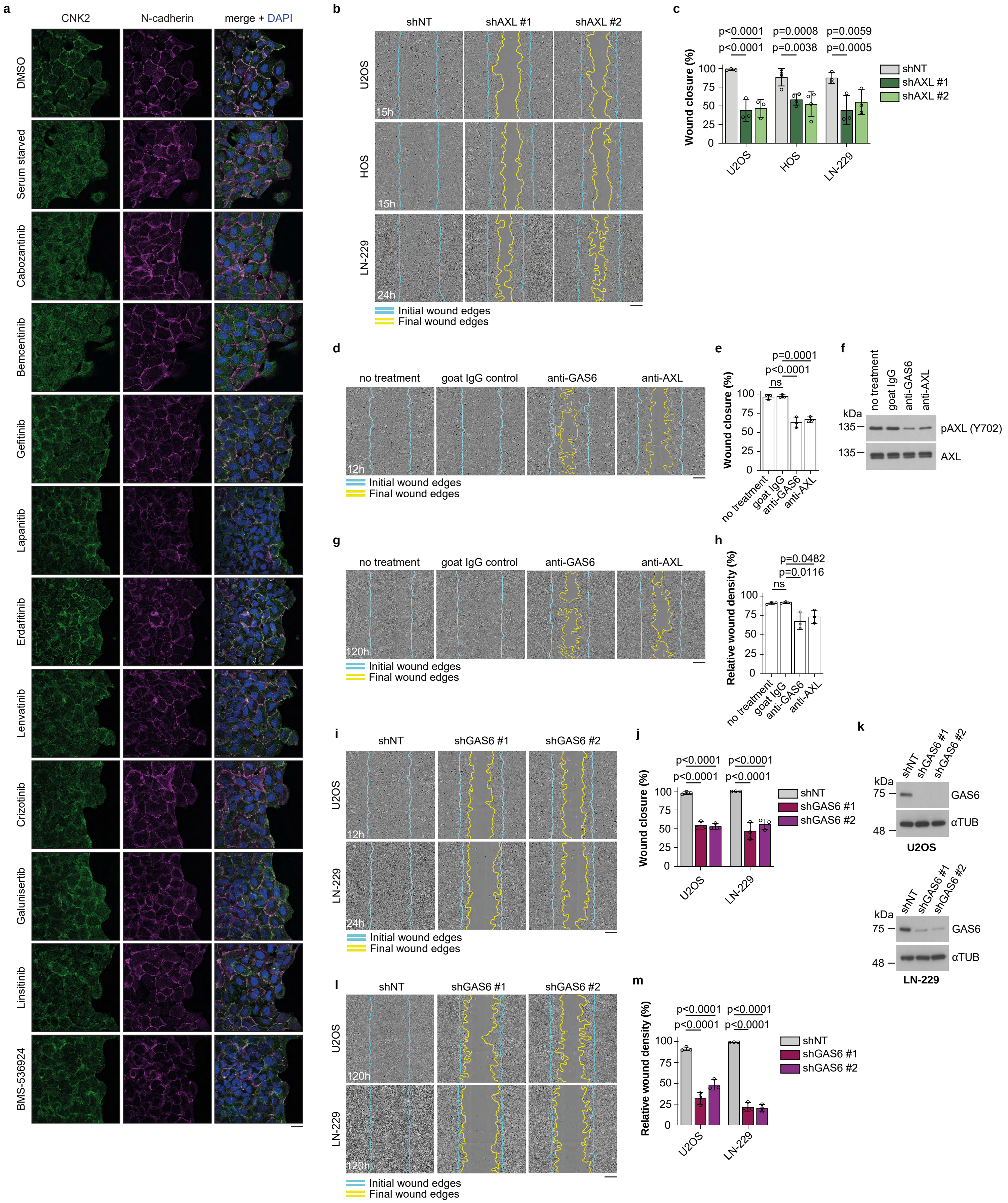
a
CNK2
N-cadherin
merge + DAPI
DMSO
Serum starved
Cabozantinib
Bemcentinib
Gefitinib
Lapanitib
Erdafitinib
Lenvatinib
Crizotinib
Galunisertib
Linsitinib
BMS-536924
b
shNT
shAXL #1
shAXL #2
U2OS
HOS
LN-229
15h
15h
24h
Initial wound edges
Final wound edges
c
p<0.0001
p<0.0001
p=0.0008
p=0.0038
p=0.0059
p=0.0005
Wound closure (%)
shNT
shAXL #1
shAXL #2
U2OS
HOS
LN-229
d
no treatment
goat IgG control
anti-GAS6
anti-AXL
12h
Initial wound edges
Final wound edges
e
p=0.0001
p<0.0001
ns
Wound closure (%)
no treatment
goat IgG
anti-GAS6
anti-AXL
f
no treatment
goat IgG
anti-GAS6
anti-AXL
kDa
135
pAXL (Y702)
135
AXL
g
no treatment
goat IgG control
anti-GAS6
anti-AXL
120h
Initial wound edges
Final wound edges
h
p=0.0482
p=0.0116
ns
Relative wound density (%)
no treatment
goat IgG
anti-GAS6
anti-AXL
i
shNT
shGAS6 #1
shGAS6 #2
U2OS
LN-229
12h
24h
Initial wound edges
Final wound edges
j
p<0.0001
p<0.0001
p<0.0001
p<0.0001
Wound closure (%)
shNT
shGAS6 #1
shGAS6 #2
U2OS
LN-229
k
shNT
shGAS6 #1
shGAS6 #2
kDa
75
GAS6
48
αTUB
U2OS
shNT
shGAS6 #1
shGAS6 #2
kDa
75
GAS6
48
αTUB
LN-229
l
shNT
shGAS6 #1
shGAS6 #2
U2OS
LN-229
120h
120h
Initial wound edges
Final wound edges
m
p<0.0001
p<0.0001
p<0.0001
p<0.0001
Relative wound density (%)
shNT
shGAS6 #1
shGAS6 #2
U2OS
LN-229

Supplementary Fig. 8: GAS6-induced AXL activation stimulates cell motility.

a Migrating U2OS cells were serum starved or treated with the indicated RTK inhibitors and stained for endogenous CNK2 and N-cadherin. **b** shRNA-mediated depletion of AXL delayed migration of U2OS, HOS and LN-229 cells in wound healing assays. **c** Quantification of the migration assays shown in **b** at the indicated time points for each cell line. **d-h** Treatment of U2OS cells with blocking antibodies against GAS6 (50 μg/mL) or AXL (20 μg/mL) reduced cell migration (**d**, **e**) and invasion (**g**, **h**) in wound healing assays and reduced AXL activation (pY702) (**f**). **i-m** GAS6 depletion with two different shRNAs (**k**) impaired cell migration (**i**, **j**) and invasion (**l**, **m**) of U2OS and LN-229 cells. All statistical analyses were performed on data from three independent experiments. Two-way ANOVA was used in **c**, **j**, **m** and one-way ANOVA was used in **e**, **h**. Error bars correspond to mean values ± SD; ns, not significant ($P>0.05$). Scale bar: (**a**) 20 μm, (**b**, **d**, **g**, **i**, **l**) 200 μm. Source data are provided as a Source Data file.

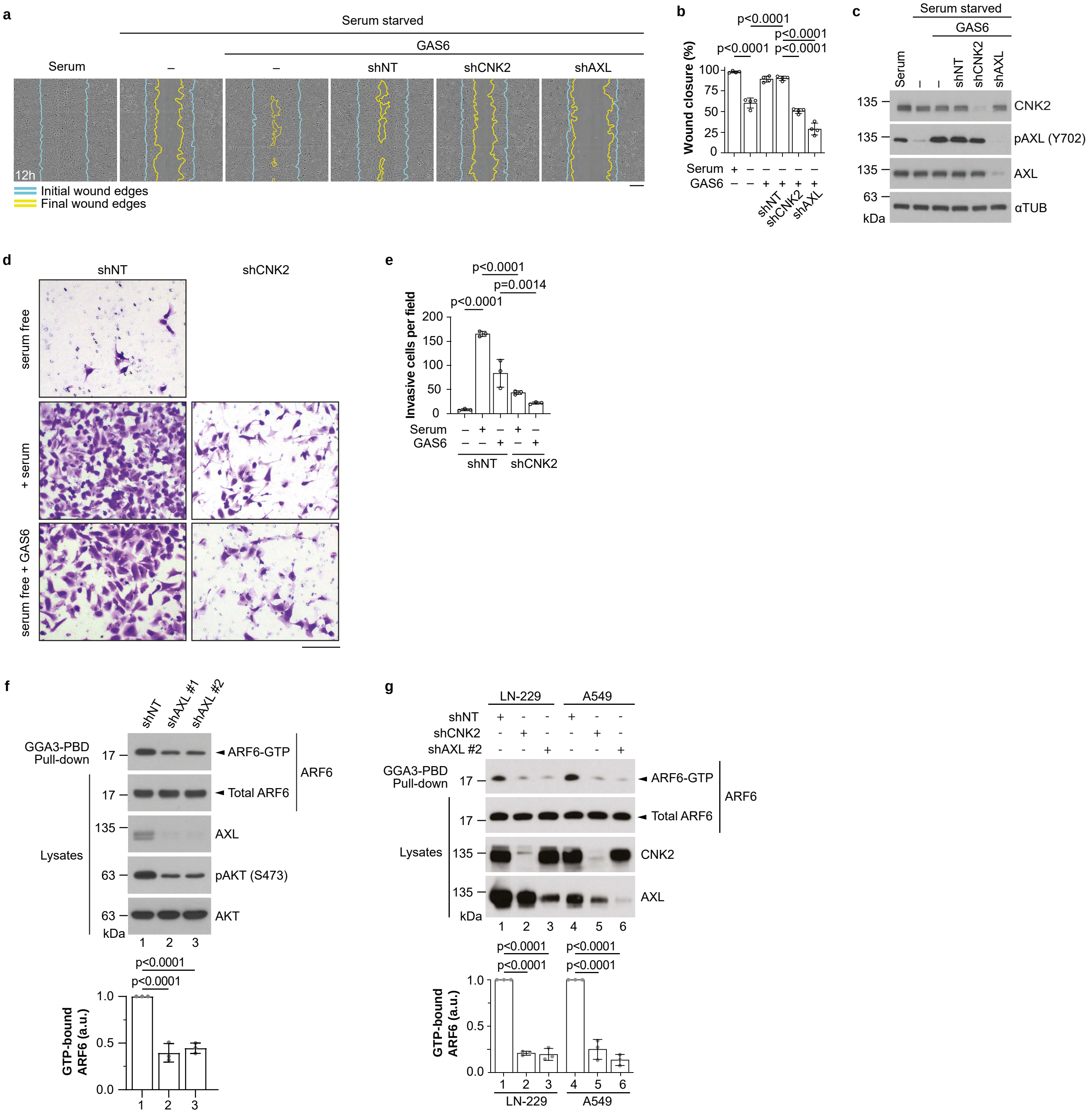

Supplementary Fig. 9: CNK2 couples AXL signalling to downstream ARF6 activation.

a-c GAS6-induced cell migration of serum starved U2OS cells was suppressed by depletion of AXL and CNK2 (**a**, **b**). Depletion of AXL or CNK2, and levels of phosphorylated AXL (pY702) are shown by western blot (**c**). **d** Invasion of U2OS cells towards 10% FBS and 200 ng/ml GAS6 in the transwell assay was strongly reduced by CNK2 depletion. **e** Quantification of the average number of cells per field for the transwell invasion assay shown in **d**. **f** Depletion of AXL in migrating U2OS cells decreased ARF6-GTP and pAKT (S473) levels. **g** Depletion of CNK2 or AXL decreased ARF6-GTP levels in migrating LN-229 and A549 cells. The bar graphs in **f** and **g** represent the normalized ratio of GTP-bound ARF6 to the amount of total ARF6. All statistical analyses were performed on data from three independent experiments. One-way ANOVA was used in **b**, **e**, **f**, and two-way ANOVA was used in **g**. Error bars correspond to mean values ± SD. Scale bar: (**a**, **d**) 200 µm. Source data are provided as a Source Data file.

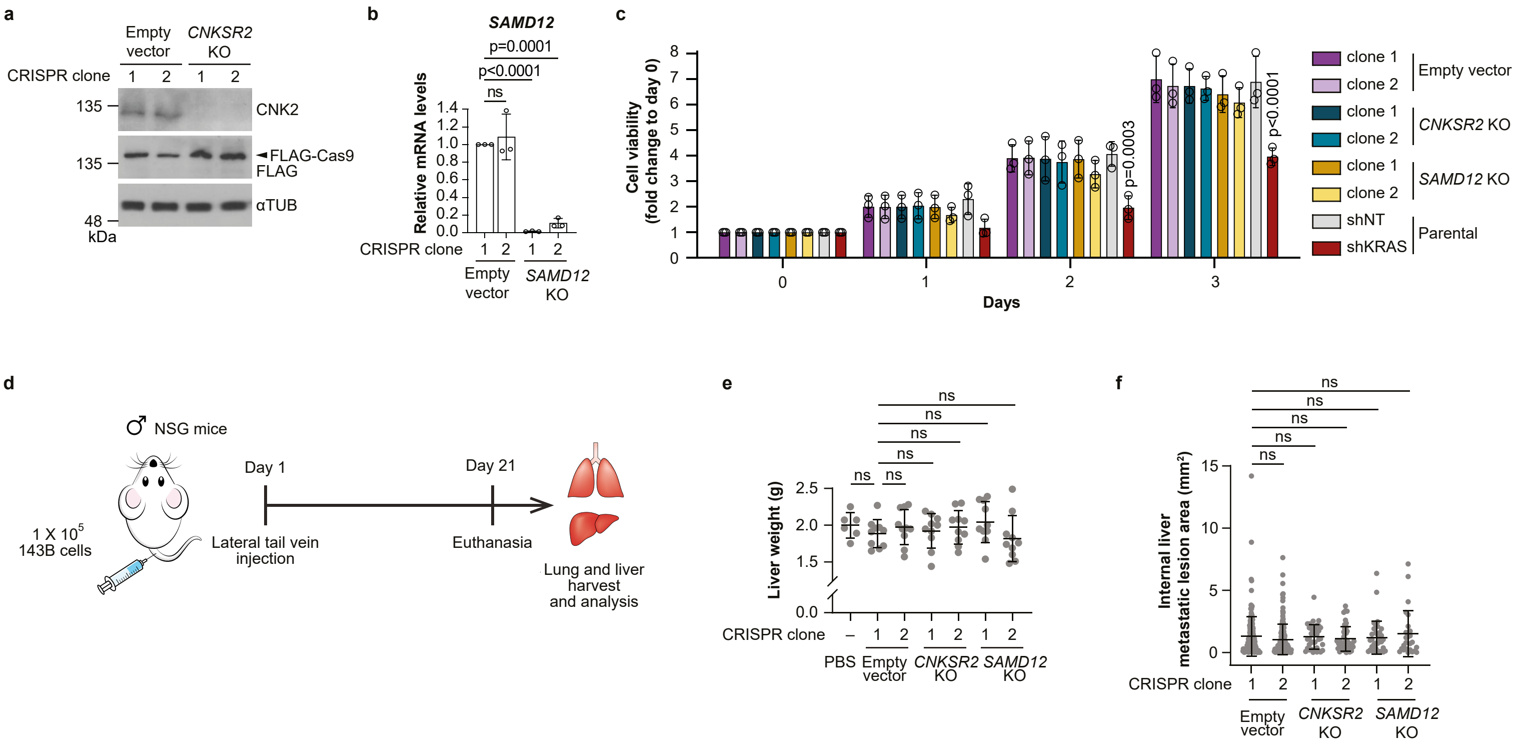

Supplementary Fig. 10: CNK2 and SAMD12 promote metastasis *in vivo*.

a Immunoblots of 143B CRISPR *CNKSR2* knockout (KO) clones. **b** *SAMD12* KO in 143B clones was quantified by RT-qPCR. **c** Neither *CNKSR2* nor *SAMD12* KO in 143B clones affected cell proliferation or viability. shRNA-mediated depletion of KRAS was used as a positive control of reduced cell proliferation or viability. **d** Schematic timeline of the *in vivo* experimental protocol. **e-f** No difference in liver weight or metastatic lesion area were observed between mice injected with control cells and $CNKSR2^{KO}$ or $SAMD12^{KO}$ cells. At the time of euthanasia, mice livers were excised and weighed (**e**). The area of tumour lesions was measured on H&E-stained liver sections (**f**). One-way ANOVA was performed on data from three independent experiments in **b**, **c** and from 6-10 mice in **e**, **f**. Error bars correspond to mean values ± SD; ns, not significant (P>0.05). Source data are provided as a Source Data file.

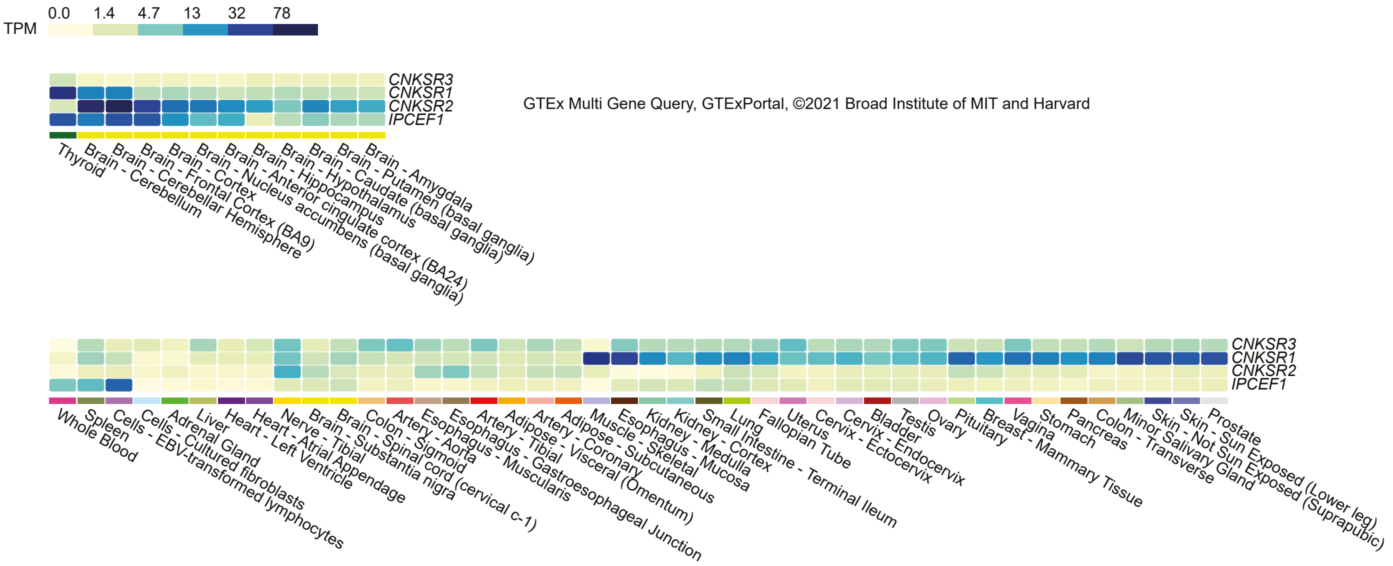

Supplementary Fig. 11: *CNKSR2* is primarily expressed in neuronal tissues.
Comparative *CNKSR1*, *CNKSR2*, *CNKSR3* and *IPCEF1* gene expression in human tissues acquired from the Genotype-Tissue Expression (GTEx) project database. Expression is in transcript per million (TPM).

Uncropped western blots from Supplementary figures

Supplementary Fig. 1a

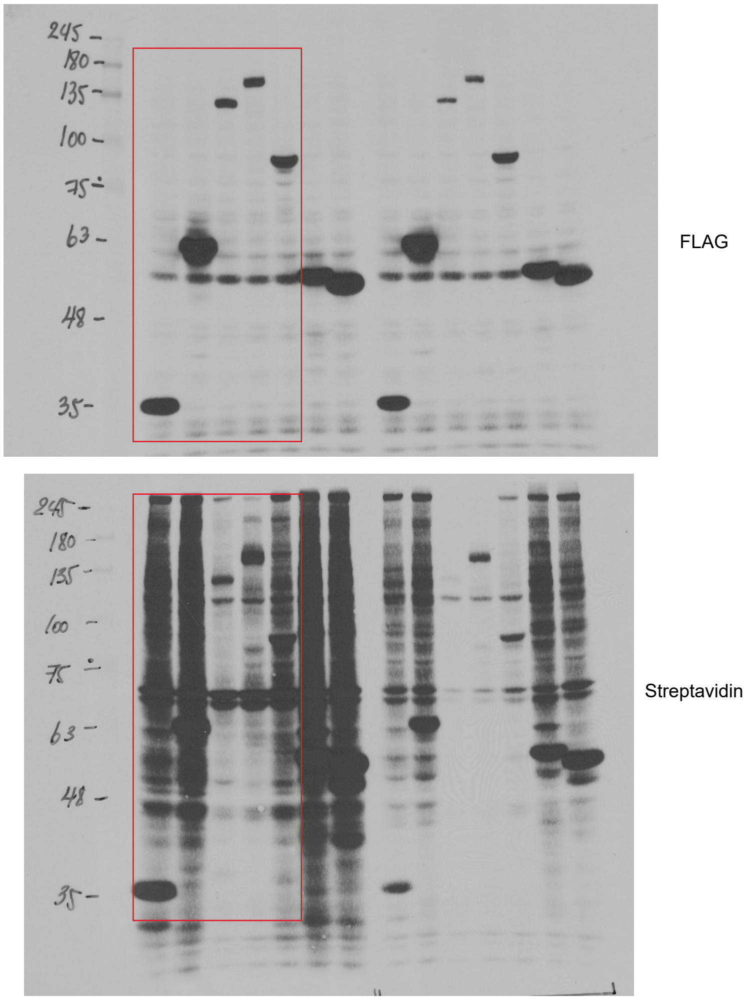

Supplementary Fig. 2c

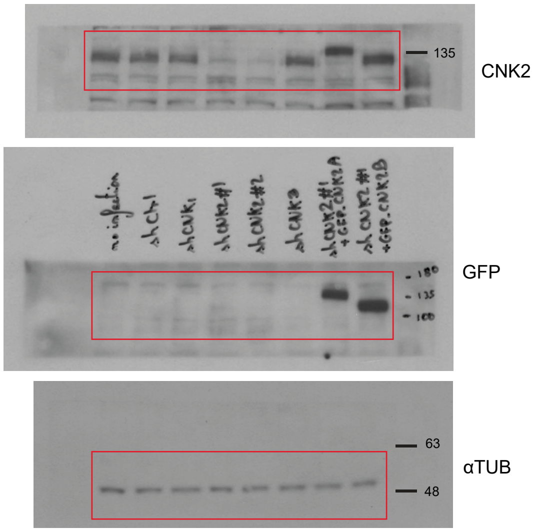

Supplementary Fig. 3a

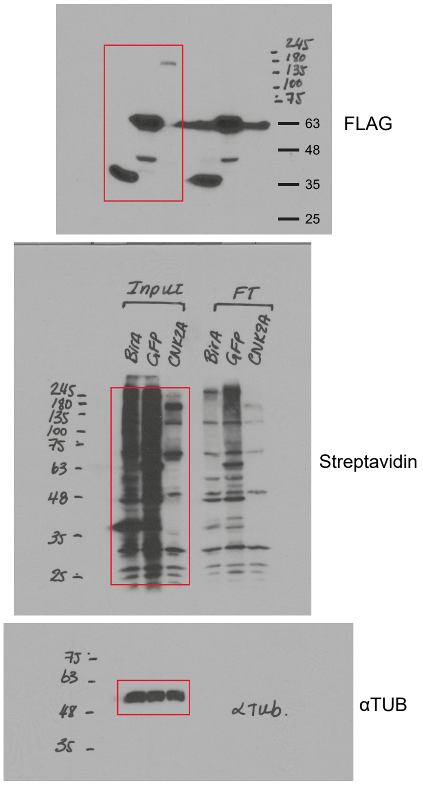

Supplementary Fig. 4a

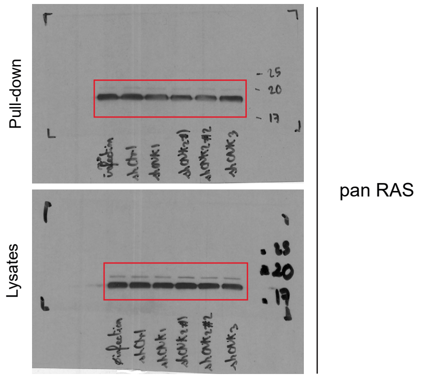

Supplementary Fig. 4h

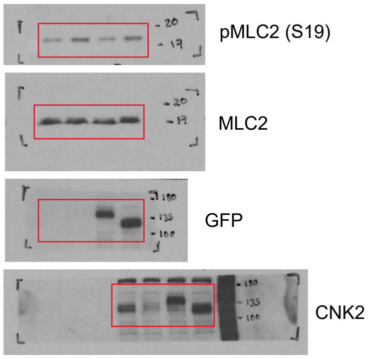

Supplementary Fig. 4i

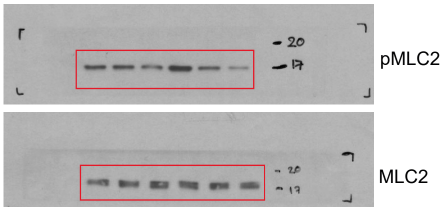

Supplementary Fig. 5a

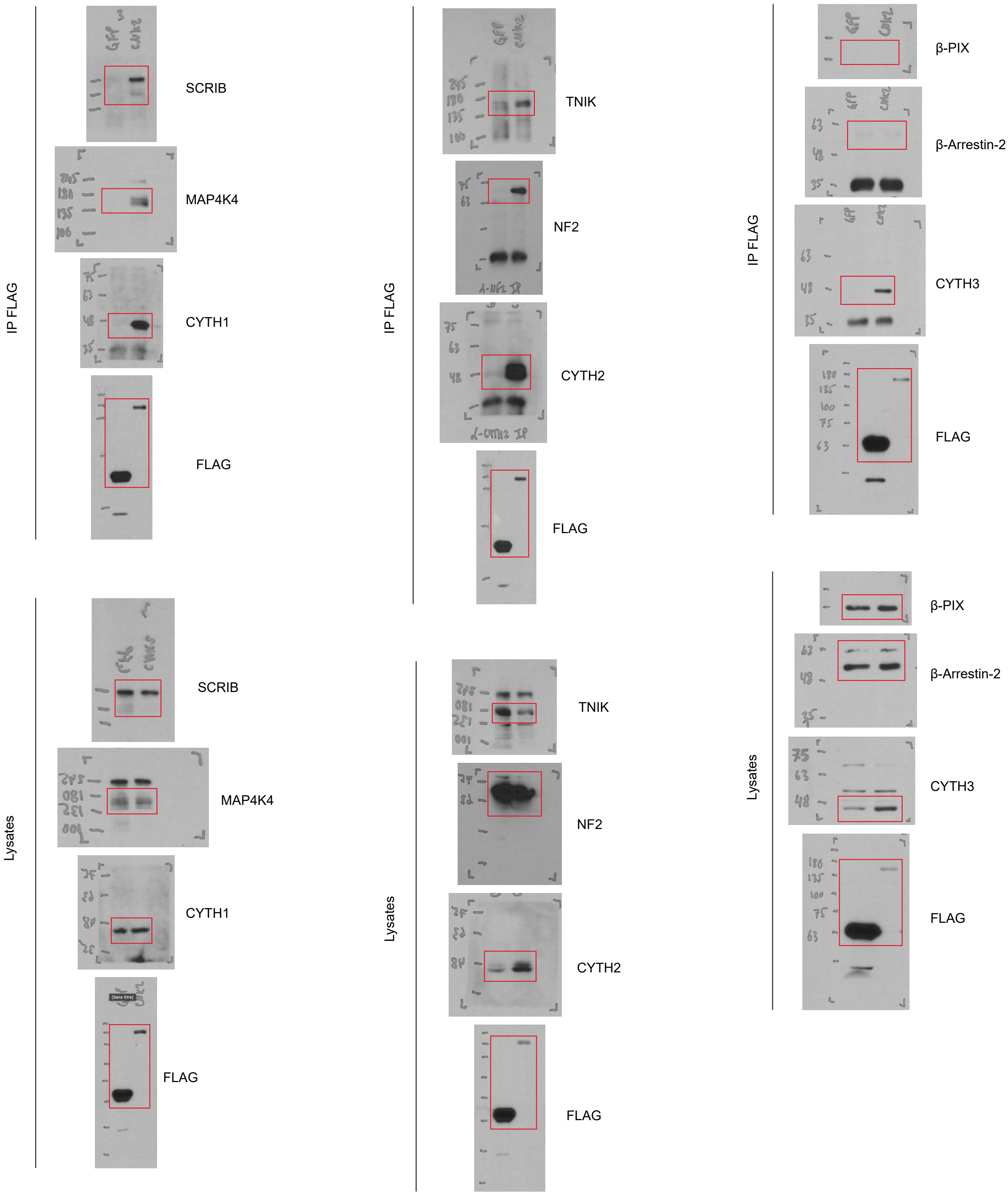

Supplementary Fig. 5b

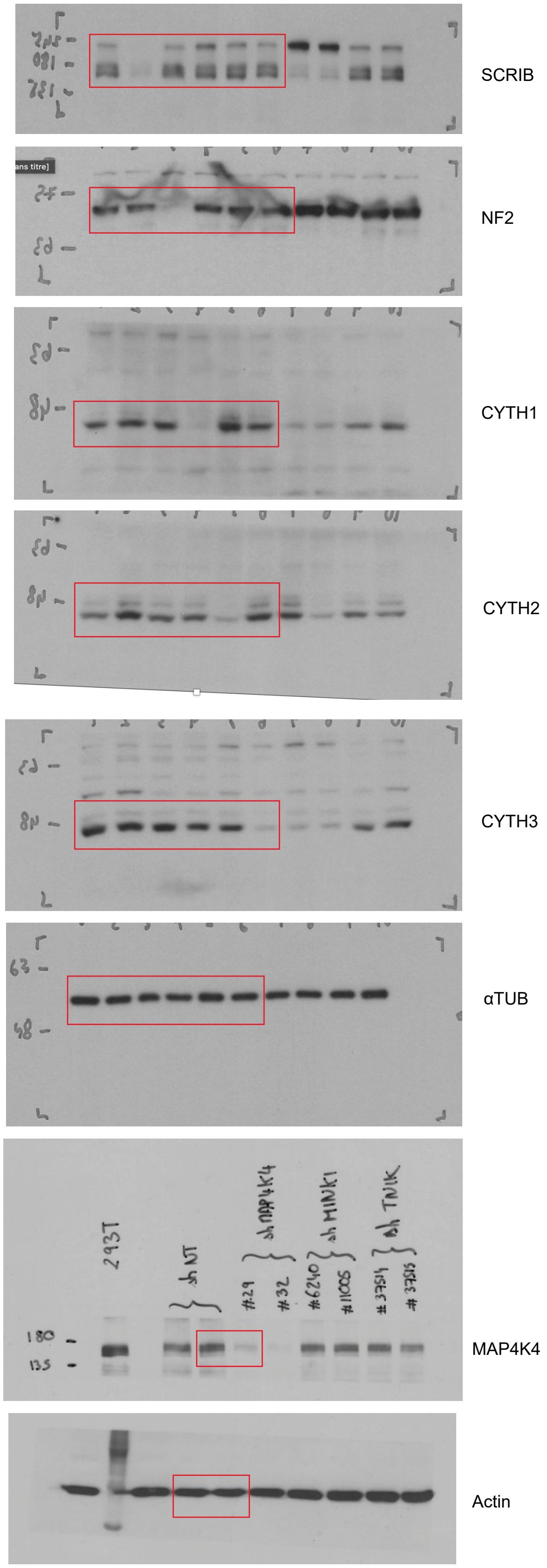

Supplementary Fig. 5d

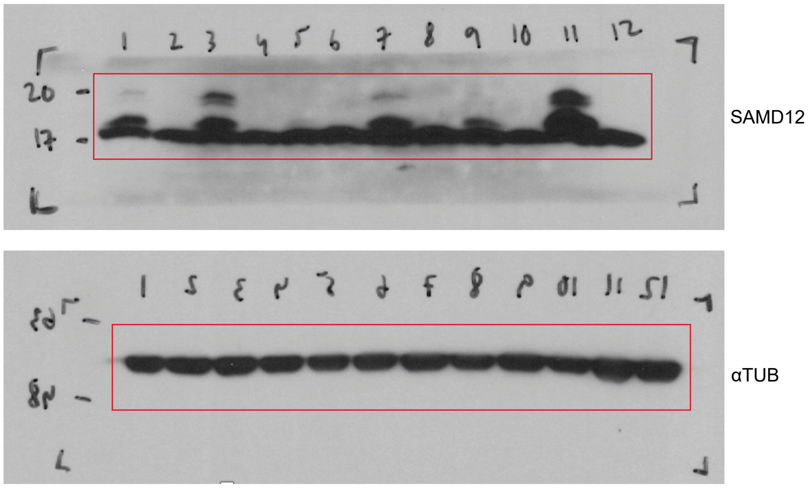

Supplementary Fig. 5e

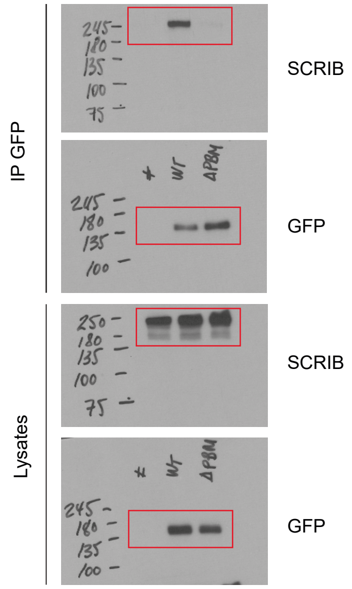

Supplementary Fig. 5f

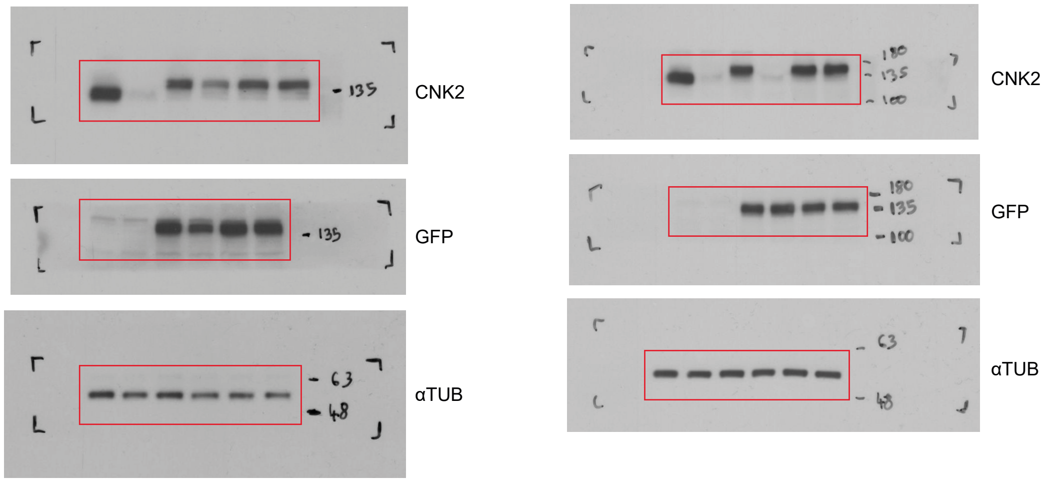

Supplementary Fig. 6a

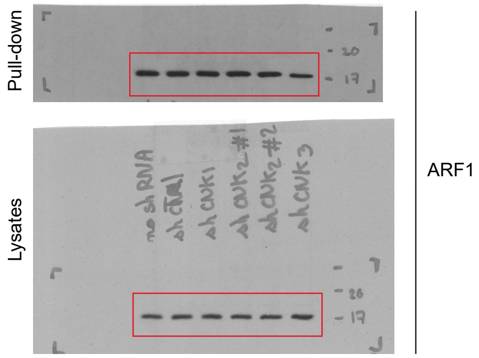

Supplementary Fig. 6e

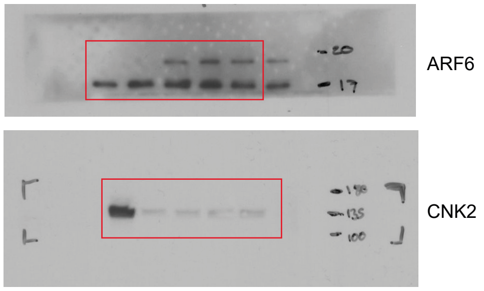

Supplementary Fig. 6d

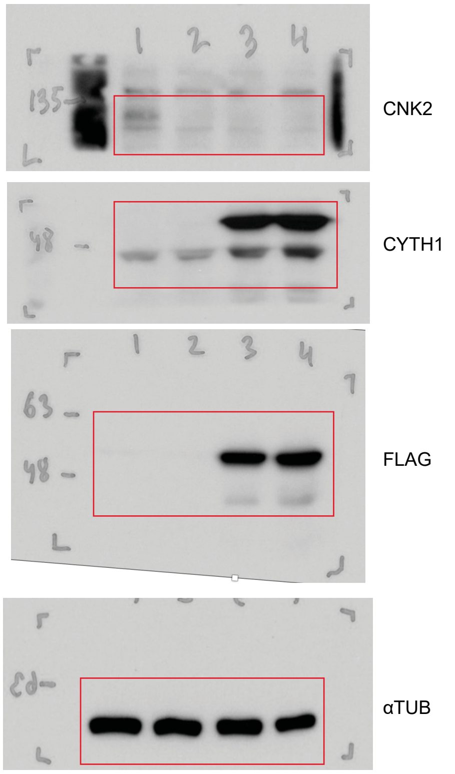

Supplementary Fig. 7b

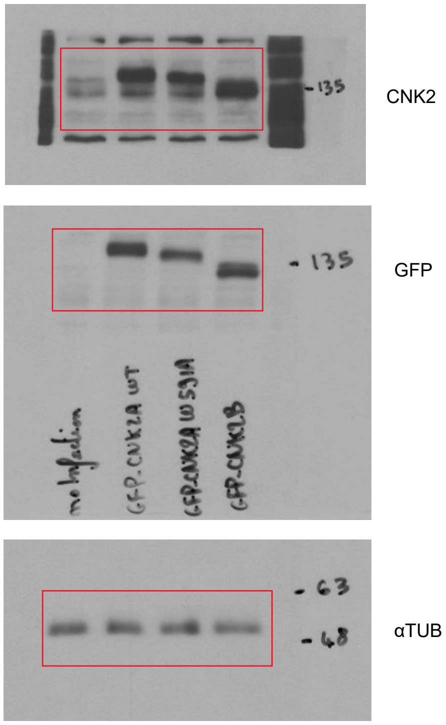

Supplementary Fig. 7j

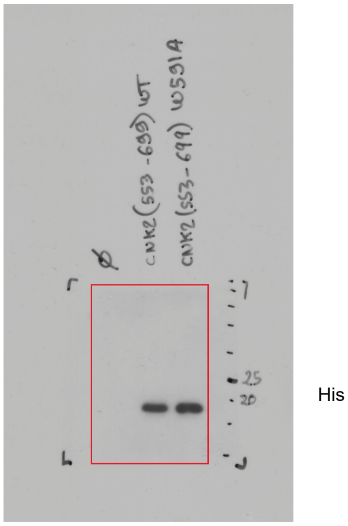

Supplementary Fig. 7m

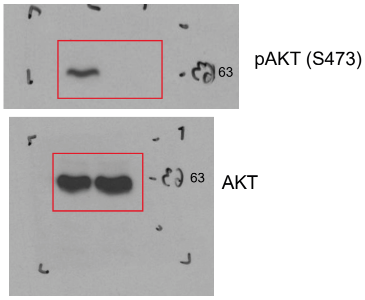

Supplementary Fig. 7n

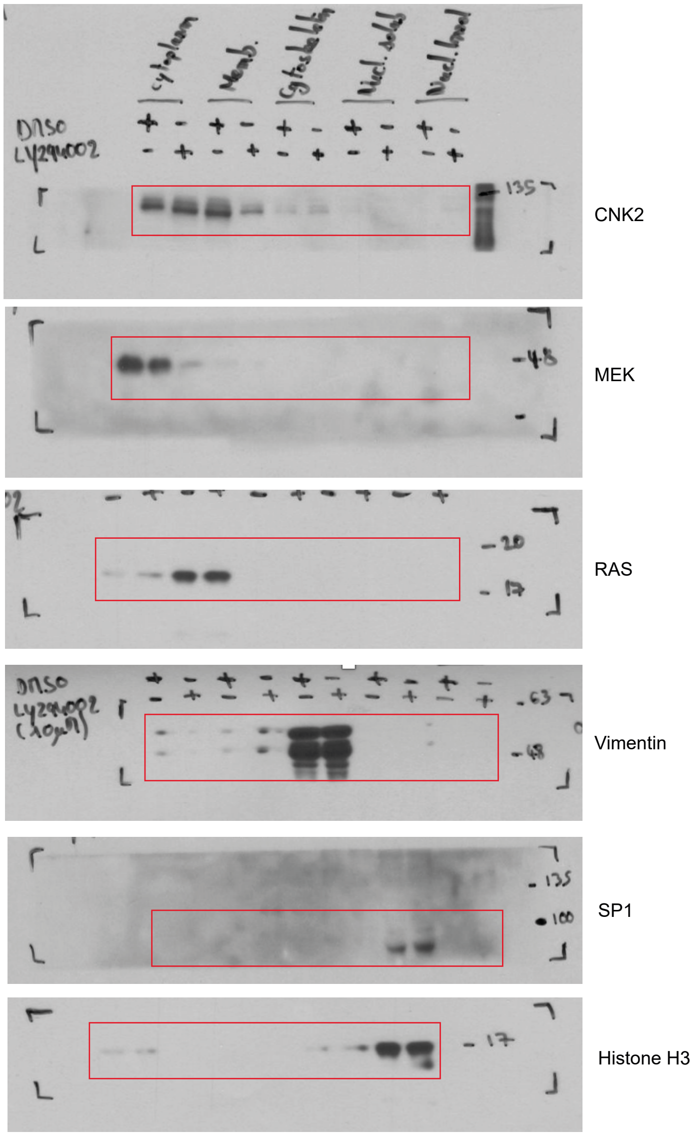

Supplementary Fig. 8f

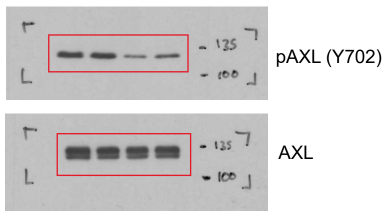

Supplementary Fig. 8k

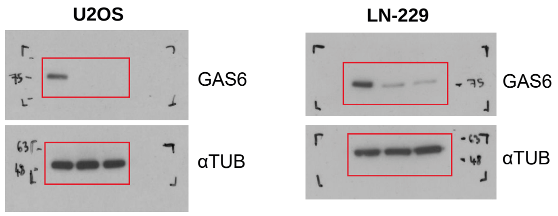

Supplementary Fig. 9c

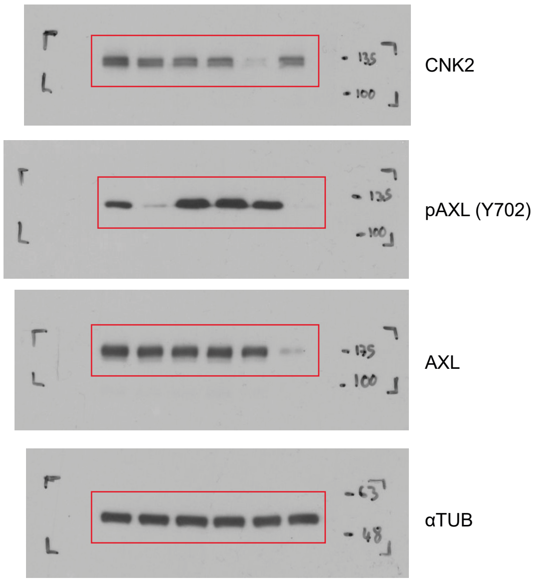

Supplementary Fig. 9f

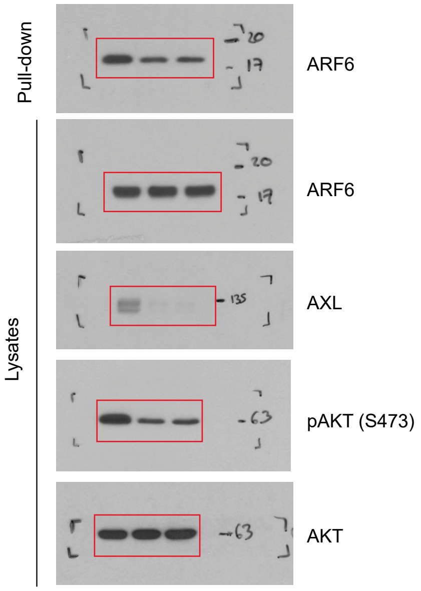

Supplementary Fig. 9g

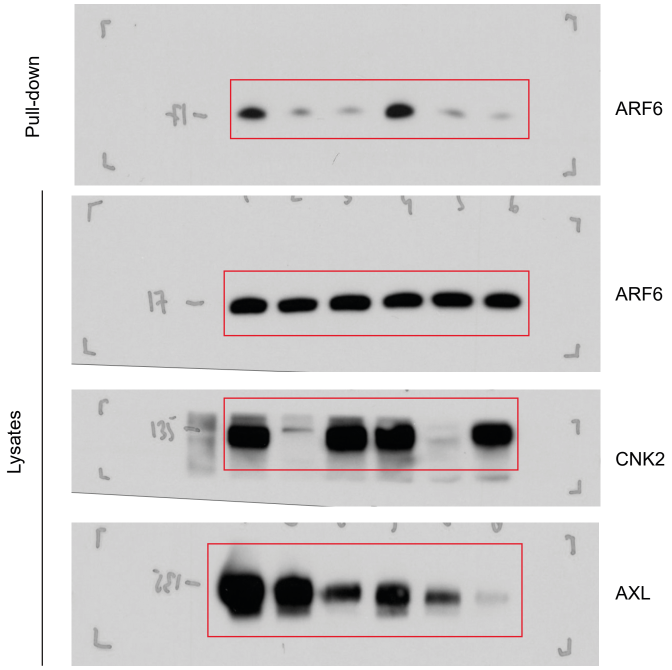

Supplementary Fig. 10a

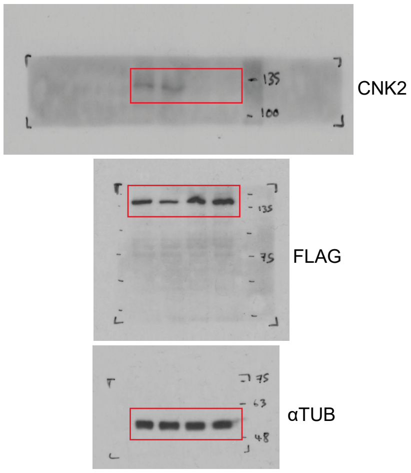

Supplement: Supplementary file 1 — Supplementary Information [file 41467_2023_39281_MOESM1_ESM.pdf]
