# Supplementary material for: CNK2 promotes cancer cell motility by mediating ARF6 activation downstream of AXL signalling
Source: Nat Commun. 2023 Jun 15;14:3560. doi: 10.1038/s41467-023-39281-z (PMC10272126; doi:10.1038/s41467-023-39281-z)
Supplement: Supplementary file 3 — Description to Additional Supplementary Information [file 41467_2023_39281_MOESM3_ESM.pdf]

Description of Additional Supplementary Files

File name: **Supplementary Data 1**
Mass spectrometry analysis of biotinylated proteins in Flp-In T-REx HEK293 cells expressing the indicated FLAG-BirA*-CNK constructs.

File name: **Supplementary Data 2**
Average RT-qPCR Ct values for CNKSR1, CNKSR2, and CNKSR3 mRNA in 39 cancer cell lines.

File name: **Supplementary Data 3**
Mass spectrometry analysis of biotinylated proteins in Flp-In T-REx U2OS cells expressing FLAG-BirA*-CNK2A.

File name: **Supplementary Data 4**
List of shRNA and oligonucleotide sequences used in this study.

File name: **Supplementary Movie 1**
CNK2A is required for the migration of U2OS cells. The migration delay observed in CNK2-depleted cells is rescued with expression of shRNA-insensitive GFP-CNK2A but not GFP-CNK2B. Time [hr:min].

File name: **Supplementary Movie 2**
The interaction of CNK2A with SAMD12 and CYTH1/3 is required for U2OS cell migration. Expression of shRNA-insensitive GFP-CNK2A_E64R/E68R or GFP-CNK2A_L955P did not rescue the migration delay observed in CNK2-depleted U2OS cells in wound healing-like assays. Time [hr:min].

File name: **Supplementary Movie 3**
Localization of CNK2A at the plasma membrane is necessary for U2OS migration. Expression of shRNA-insensitive GFP-CNK2A_W591A did not rescue the migration delay observed in CNK2-depleted U2OS cells in wound healing-like assays. Time [hr:min].

File name: **Supplementary Movie 4**
CNK2A functions at the plasma membrane. Overexpression of GFP-CNK2A WT, but not GFP-CNK2A_W591A or GFP-CNK2B, increased the migration speed of U2OS cells in wound healing-like assays. Time [hr:min].
