# Supplementary material for: CNK2 promotes cancer cell motility by mediating ARF6 activation downstream of AXL signalling
Source: Nat Commun. 2023 Jun 15;14:3560. doi: 10.1038/s41467-023-39281-z (PMC10272126; doi:10.1038/s41467-023-39281-z)
Supplement: Supplementary file 12 — Reporting Summary [file 41467_2023_39281_MOESM12_ESM.pdf]

nature portfolio

Corresponding author(s): Marc Therrien

Last updated by author(s): 2023/04/21

Reporting Summary

Nature Portfolio wishes to improve the reproducibility of the work that we publish. This form provides structure for consistency and transparency in reporting. For further information on Nature Portfolio policies, see our Editorial Policies and the Editorial Policy Checklist.

Statistics

For all statistical analyses, confirm that the following items are present in the figure legend, table legend, main text, or Methods section.

n/a	Confirmed	
☐	☒	The exact sample size (*n*) for each experimental group/condition, given as a discrete number and unit of measurement
☐	☒	A statement on whether measurements were taken from distinct samples or whether the same sample was measured repeatedly
☐	☒	The statistical test(s) used AND whether they are one- or two-sided<br>*Only common tests should be described solely by name; describe more complex techniques in the Methods section.*
☒	☐	A description of all covariates tested
☒	☐	A description of any assumptions or corrections, such as tests of normality and adjustment for multiple comparisons
☐	☒	A full description of the statistical parameters including central tendency (e.g. means) or other basic estimates (e.g. regression coefficient) AND variation (e.g. standard deviation) or associated estimates of uncertainty (e.g. confidence intervals)
☐	☒	For null hypothesis testing, the test statistic (e.g. *F*, *t*, *r*) with confidence intervals, effect sizes, degrees of freedom and *P* value noted<br>*Give P values as exact values whenever suitable.*
☒	☐	For Bayesian analysis, information on the choice of priors and Markov chain Monte Carlo settings
☒	☐	For hierarchical and complex designs, identification of the appropriate level for tests and full reporting of outcomes
☒	☐	Estimates of effect sizes (e.g. Cohen's *d*, Pearson's *r*), indicating how they were calculated

Our web collection on statistics for biologists contains articles on many of the points above.

Software and code

Policy information about availability of computer code

Data collection	ImageJ software was version 2.1.0/1.53d and the Ibidi chemotaxis tool was version 2.0. “MRI wound healing tool” and "Manual tracking" plugins for ImageJ were used. Zen Blue was version 3.0 and Zen Black (2011) was version 1.1.1.0. Imaris software was version 9.1.2. Incucyte S3 software was version 2020B and 2021C. For mass spectrometry and BioID data: PEAKS was version X Pro, Scaffold Viewer software was version 5.1.2, and the express version of SAINT was used. NDP.view2Plus software was version 2.9.29. Expression Suite was version 1.3.
Data analysis	GraphPad Prism 9.0 was used for data analysis.

For manuscripts utilizing custom algorithms or software that are central to the research but not yet described in published literature, software must be made available to editors and reviewers. We strongly encourage code deposition in a community repository (e.g. GitHub). See the Nature Portfolio guidelines for submitting code & software for further information.

Data

Policy information about availability of data

All manuscripts must include a data availability statement. This statement should provide the following information, where applicable:

- Accession codes, unique identifiers, or web links for publicly available datasets
- A description of any restrictions on data availability
- For clinical datasets or third party data, please ensure that the statement adheres to our policy

Data availability

Publicly available Gene Ontology terms used to classify BioID hits (Supplementary Fig. 1e; Supplementary Fig. 3d) are available on STRING (https://string-db.org/). Comparative expression of CNKSR1, CNKSR2, CNKSR3 and IPCEF1 genes in healthy human tissues were acquired from the Genotype-Tissue Expression (GTEx) database on 02/01/22 using the Multi-Gene Query expression option (https://gtexportal.org/home/). The mass spectrometry proteomics data generated in this study (Supplementary Data 1 and 3) are available via the ProteomeXchange Consortium through the PRIDE partner repository under the accession code PXD038687 (http://www.ebi.ac.uk/pride/archive/projects/PXD038687). The remaining data are available within the Article, Supplementary Information or Source Data files. Source data are provided with this paper.

Human research participants

Policy information about studies involving human research participants and Sex and Gender in Research.

Reporting on sex and gender	This study did involve human research participants or Sex and Gender in Research.
Population characteristics	N/A.
Recruitment	N/A.
Ethics oversight	N/A.

Note that full information on the approval of the study protocol must also be provided in the manuscript.

Field-specific reporting

Please select the one below that is the best fit for your research. If you are not sure, read the appropriate sections before making your selection.

☒ Life sciences ☐ Behavioural & social sciences ☐ Ecological, evolutionary & environmental sciences

For a reference copy of the document with all sections, see nature.com/documents/nr-reporting-summary-flat.pdf

Life sciences study design

All studies must disclose on these points even when the disclosure is negative.

Sample size	Samples sizes were determined based on standard practices in the field (Lazic et al., 2018; doi: 10.1371/journal.pbio.2005282). Please refer to the "statistics and reproducibility" Methods section, other Methods sections for details of specific experiments, and Figure legends.
Data exclusions	No data was excluded.
Replication	Statistical tests were used to determine the reproducibility of the experiments. We used at least three independent experiments or six biologically independent samples (mice) for statistical analysis. PIP Strip assays, subcellular fractionations, and immunoblots or confocal images without associated statistical analysis were conducted at least twice with reproducible results. All other experiments in the manuscript were performed at least three times independently, with similar results.
Randomization	Treatments with inhibitors, lentiviral infections, and transfections were randomly administered to cell populations that were otherwise cultured identically prior to the experiments. For in vivo experiments, individual mice were randomly selected for injection of different knockout cell lines.
Blinding	Investigators were not blinded. Unless stated otherwise, all data collection was done using the softwares indicated above. The use of ImageJ, Imaris, and Incucyte automated analyses reduced potential user bias.

Reporting for specific materials, systems and methods

We require information from authors about some types of materials, experimental systems and methods used in many studies. Here, indicate whether each material, system or method listed is relevant to your study. If you are not sure if a list item applies to your research, read the appropriate section before selecting a response.

Materials & experimental systems

n/a	Involved in the study
☐	☒ Antibodies
☐	☒ Eukaryotic cell lines
☒	☐ Palaeontology and archaeology
☐	☒ Animals and other organisms
☒	☐ Clinical data
☒	☐ Dual use research of concern

Methods

n/a	Involved in the study
☒	☐ ChIP-seq
☒	☐ Flow cytometry
☒	☐ MRI-based neuroimaging

Antibodies

Antibodies used

The following antibodies were used for western blotting: anti-CNK2 from rabbit (1:500; Cedarlane Laboratories; custom-made antibody), anti-α-Tubulin DM1A from mouse (1:10,000; Millipore Sigma; #T9026), anti-CNKSR3 (CNK3) from mouse (1:1000; Abnova; #H00154043), anti-SAMD12 (A-6) from mouse (1:500; Santa Cruz Biotechnology, Inc.; #sc-377123), anti-FLAG M2 from mouse (1:5000; Sigma-Aldrich; #F1804), anti-GFP from rabbit (1:5000; OriGene; #TP401), anti-Phospho-Myosin Light Chain 2 (S19) from rabbit (1:500; Cell Signaling Technology; #3671), anti-Myosin Light Chain 2 from rabbit (1:1000; Cell Signaling Technology; #3672), anti-HGK (MAP4K4) from rabbit (1:1000; Cell Signaling Technology; #3485), anti-TNIK from rabbit (1:1000; GeneTex; #GTX13141), anti-NF2 from rabbit (1:1000; Sigma-Aldrich; #HPA003097), anti-Cytohesin 1 (2E11) (CYTH1) from mouse (1:500; Invitrogen; #MA1-060), anti-Cytohesin-2 (H-7) (CYTH2) from mouse (1:250; Santa Cruz Biotechnology, Inc.; #sc-374640), anti-Cytohesin 3 (CYTH3) from rabbit (1:1000; Abcam; #ab155691); anti-Scribble (SCRIB) from rabbit (1:1000; Cell Signaling Technology; #4475), anti-Cool1/βPix from rabbit (1:1000; Cell Signaling Technology; #4515), anti-β-Arrestin 2 (C16D9) from rabbit (1:1000; Cell Signaling Technology; #3857), anti-RhoA from mouse (1:1000; Cytoskeleton, Inc.; #ARH04), anti-Rac1 from mouse (1:500; Cytoskeleton, Inc.; #ARC03), anti-Cdc42 from mouse (1:500; Cytoskeleton, Inc.; #ACD03), anti-Arf1 from mouse (1:500; Cytoskeleton, Inc.; #ARF01), anti-Arf6 from mouse (1:500; Cytoskeleton, Inc.; #ARF06), anti-Pan Ras from mouse (1:1000; Cytoskeleton, Inc.; #AESA02), anti-Axl (C89E7) from rabbit (1:1000; Cell Signaling Technology; #8661), anti-phospho-Axl (Tyr702) (D12B2) from rabbit (1:1000; Cell Signaling Technology; #5724), anti-GAS6 (D3A3G) from rabbit (1:1000; Cell Signaling Technology; #67202), anti-Akt (pan) (C67E7) from rabbit (1:2000; Cell Signaling Technology; #4691), anti-phospho-Akt (Ser473) (D9E) from rabbit (1:2000; Cell Signaling Technology; #4060), anti-Vimentin (D21H3) from rabbit (1:10,000; Cell Signaling Technology; #5741), anti-SP1 (D4C3) from rabbit (1:1000; Cell Signaling Technology; #9389), anti-MEK1/2 from rabbit (1:2000; Cell Signaling Technology; #9122), anti-Histone H3 (3H1) from rabbit (1:1000; Cell Signaling Technology; #9717), anti-V5 Tag from mouse (1:5000; Invitrogen; #R960-25), anti-His-probe (H15) from rabbit (1:1000; Santa Cruz Biotechnology, Inc.; #sc-803), and anti-Actin (C4) from mouse (1:5000; Millipore Sigma; #MAB1501).

The following antibodies were used for immunofluorescence: anti-CNK2 produced in rabbit (1:100; Cedarlane Laboratories; custom-made antibody), anti-Phospho-Myosin Light Chain 2 (S19) from rabbit (1:200; Cell Signaling Technology; #3671), anti-Zyxin (2D1) from mouse (1:300; Santa Cruz Biotechnology, Inc.; #sc-293448), anti-N-Cadherin from mouse (1:300; BD Transduction Laboratories; #610921), anti-GFP from rabbit (1:500; OriGene; #TP401) and anti-Flag M2 from mouse (1:1000; Sigma-Aldrich; #F1804).

Validation

The following antibodies were validated in our study using shRNA and western blotting: anti-CNK2 from rabbit (Cedarlane Laboratories; custom-made antibody), anti-CNKSR3 (CNK3) from mouse (Abnova; #H00154043), anti-SAMD12 (A-6) from mouse (Santa Cruz Biotechnology, Inc.; #sc-377123), anti-HGK (MAP4K4) from rabbit (Cell Signaling Technology; #3485), anti-Cytohesin 1 (2E11) (CYTH1) from mouse (Invitrogen; #MA1-060), anti-Cytohesin-2 (H-7) (CYTH2) from mouse (Santa Cruz Biotechnology, Inc.; #sc-374640), anti-Cytohesin 3 (CYTH3) from rabbit (Abcam; #ab155691); anti-Scribble (SCRIB) from rabbit (Cell Signaling Technology; #4475), anti-RhoA from mouse (Cytoskeleton, Inc.; #ARH04), anti-Rac1 from mouse (Cytoskeleton, Inc.; #ARC03), anti-Arf6 from mouse (Cytoskeleton, Inc.; #ARF06), anti-Axl (C89E7) from rabbit (Cell Signaling Technology; #8661), anti-GAS6 (D3A3G) from rabbit (Cell Signaling Technology; #67202), anti-NF2 from rabbit (Sigma-Aldrich; #HPA003097).

The following antibodies were validated in our study using recombinant ligands or inhibitors, and western blotting: anti-phospho-Axl (Tyr702) (D12B2) from rabbit (Cell Signaling Technology; #5724), anti-phospho-Akt (Ser473) (D9E) from rabbit (Cell Signaling Technology; #4060).

The following antibodies were validated in our study through exogenous expression of tagged recombinant proteins: anti-FLAG M2 from mouse (Sigma-Aldrich; #F1804), anti-GFP from rabbit (OriGene; #TP401), anti-His-probe (H15) from rabbit (Santa Cruz Biotechnology, Inc.; #sc-803), anti-V5 Tag from mouse (Invitrogen; #R960-25).

The following antibodies were used in previous studies or validated by manufacturers: anti-α-Tubulin DM1A from mouse (Millipore Sigma; #T9026) was validated by the manufacturer using independent antibodies recognizing a different epitope of the protein (see manufacturer datasheet). Anti-Phospho-Myosin Light Chain 2 (S19) from rabbit (Cell Signaling Technology; #3671) was validated using a myosin light chain kinase inhibitor (see manufacturer datasheet). Anti-Myosin Light Chain 2 from rabbit (Cell Signaling Technology; #3672) was used in Kwon et al., Nat Comm 13:7617 (2022). Anti-Cool1/βPix from rabbit (Cell Signaling Technology; #4515) was used in Masi et al., Cell Rep 34:108800 (2021). Anti-β-Arrestin 2 (C16D9) from rabbit (Cell Signaling Technology; #3857) was used in Kawakami et al., Nat Comm 13:487 (2022). Anti-Cdc42 from mouse (Cytoskeleton, Inc.; #ACD03), anti-Arf1 from mouse (Cytoskeleton, Inc.; #ARF01) and anti-Pan Ras from mouse (Cytoskeleton, Inc.; #AESA02) were validated by the manufacturer using recombinant proteins. Anti-Akt (pan) (C67E7) from rabbit (Cell Signaling Technology; #4691) was used in Lekka et al., Nat Comm 13:7940 (2022). Anti-Vimentin (D21H3) from rabbit (Cell Signaling Technology; #5741) was validated using a knockout cell line (see manufacturer datasheet). Anti-SP1 (D4C3) from rabbit (Cell Signaling Technology; #9389) was used in Verma et al., Nat Comm 13:7344 (2022). Anti-MEK1/2 from rabbit (Cell Signaling Technology; #9122) was used in Simmler et al., Cell Rep 40:111266 (2022). Anti-Histone H3 (3H1) from rabbit (Cell Signaling Technology; #9717) was used in Asada-Utsugi et al., Commun Biol. 5:358 (2022). Anti-Actin (C4) from mouse (Millipore Sigma; #MAB1501) was used in Tumbale et al., Nature 506:111-115 (2013). Anti-Zyxin (2D1) from mouse (Santa Cruz Biotechnology, Inc.; #sc-293448) was used in Yan et al., Cell Death Dis 12:955 (2021). Anti-N-Cadherin from

	mouse (BD Transduction Laboratories; #610921) was used in Izawa et al., J Biol Chem. 277:5345-5350 (2002). Anti-TNIK from rabbit (GeneTex; #GTX13141) was validated using shRNA (see manufacturer datasheet).

Eukaryotic cell lines

Policy information about cell lines and Sex and Gender in Research

Cell line source(s)	Please refer to the "cell culture" Methods section and Table S2 for the origins of all cell lines used.
Authentication	Cell lines were not authenticated.
Mycoplasma contamination	All cell lines tested negative for mycoplasma.
Commonly misidentified lines (See ICLAC register)	None of the 7 cell lines that we used in functional experiments (U2OS, HOS, A549, A375, LN-229, MDA-MB-231, 143B) are categorized as "misidentified cell lines" in the ICLAC register.

Animals and other research organisms

Policy information about studies involving animals; ARRIVE guidelines recommended for reporting animal research, and Sex and Gender in Research

Laboratory animals	Immunodeficient NOD.Cg-Prkdcscid Il2rgtm1Wjl/SzJ mice (NSG) were obtained from Jackson Laboratory (RRID:IMSR_JAX:005557). Mice were housed under specific pathogen-free conditions in ventilated racks within filter-topped isolator cages, with a 12/12 h light/dark cycle, at constant temperature (20°C +/- 2°C) and humidity (50% +/- 10%), and with access to food and water ad libitum. We used 14-17 week-old male NSG mice.
Wild animals	The study did not involve wild animals.
Reporting on sex	This information has not been collected.
Field-collected samples	The study did not involve samples collected from the fields.
Ethics oversight	All experiments involving mice were approved by the Université de Montréal Institutional Animal Care Committee in compliance with guidelines from the Canadian Council on Animal Care.

Note that full information on the approval of the study protocol must also be provided in the manuscript.
